# Global prevalence and effect of comorbidities and smoking status on severity and mortality of COVID-19 in association with age and gender: a systematic review, meta-analysis and meta-regression

**DOI:** 10.1038/s41598-023-33314-9

**Published:** 2023-04-19

**Authors:** Santenna Chenchula, Kota Vidyasagar, Saman Pathan, Sushil Sharma, Madhav Rao Chavan, Akshaya Srikanth Bhagavathula, R. Padmavathi, M. Manjula, Manik Chhabra, Rupesh Gupta, Krishna Chaitanya Amerneni, Mohan Krishna Ghanta, Sofia Mudda

**Affiliations:** 1 Department of Pharmacology, All India Institute of Medical Sciences (AIIMS), Mangalagiri, Andhra Pradesh 522503 India; 2 Department of Pharmaceutical Sciences, University College of Pharmaceutical Sciences (UCPSc), Hanmakonda, Telangana India; 3 Department of Pharmacology, All India Institute of Medical Sciences, Bhopal, India; 4 Center for public health and technology, University of Arkansas, Fayetteville, Arkansas US; 5 SVS Medical College and Hospital, Mahbubnagar, Telangana India; 6 Balaji College of Nursing, Tirupathi, Andhra Pradesh India; 7 Department of Pharmacy Practice, Indo-Soviet Friendship College of Pharmacy, Moga, India; 8 Department of Internal Medicine, GMC, Shahdol, Madhya Pradesh India; 9 Department of Internal Medicine, Western Michigan University, Kalamazoo, USA; 10 Department of Pharmacology, MVJ Medical College, Hoskote, Karnataka India; 11 Department of AYUSH, All India Institute of Medical Sciences, Bhopal, India

**Keywords:** Diseases, Health care, Medical research, Risk factors

## Abstract

A COVID-19 patient often presents with multiple comorbidities and is associated with adverse outcomes. A comprehensive assessment of the prevalence of comorbidities in patients with COVID-19 is essential. This study aimed to assess the prevalence of comorbidities, severity and mortality with regard to geographic region, age, gender and smoking status in patients with COVID-19. A systematic review and multistage meta-analyses were reported using PRISMA guidelines. PubMed/MEDLINE, SCOPUS, Google Scholar and EMBASE were searched from January 2020 to October 2022. Cross-sectional studies, cohort studies, case series studies, and case–control studies on comorbidities reporting among the COVID-19 populations that were published in English were included. The pooled prevalence of various medical conditions in COVID-19 patients was calculated based on regional population size weights. Stratified analyses were performed to understand the variations in the medical conditions based on age, gender, and geographic region. A total of 190 studies comprising 105 million COVID-19 patients were included. Statistical analyses were performed using STATA software, version 16 MP (StataCorp, College Station, TX). Meta-analysis of proportion was performed to obtain pooled values of the prevalence of medical comorbidities: hypertension (39%, 95% CI 36–42, n = 170 studies), obesity (27%, 95% CI 25–30%, n = 169 studies), diabetes (27%, 95% CI 25–30%, n = 175), and asthma (8%, 95% CI 7–9%, n = 112). Moreover, the prevalence of hospitalization was 35% (95% CI 29–41%, n = 61), intensive care admissions 17% (95% CI 14–21, n = 106), and mortality 18% (95% CI 16–21%, n = 145). The prevalence of hypertension was highest in Europe at 44% (95% CI 39–47%, n = 68), obesity and diabetes at 30% (95% CI, 26–34, n = 79) and 27% (95%CI, 24–30, n = 80) in North America, and asthma in Europe at 9% (95% CI 8–11, n = 41). Obesity was high among the ≥ 50 years (30%, n = 112) age group, diabetes among Men (26%, n = 124) and observational studies reported higher mortality than case–control studies (19% vs. 14%). Random effects meta-regression found a significant association between age and diabetes (*p* < 0.001), hypertension (*p* < 0.001), asthma (*p* < 0.05), ICU admission (*p* < 0.05) and mortality (*p* < 0.001). Overall, a higher global prevalence of hypertension (39%) and a lower prevalence of asthma (8%), and 18% of mortality were found in patients with COVID-19. Hence, geographical regions with respective chronic medical comorbidities should accelerate regular booster dose vaccination, preferably to those patients with chronic comorbidities, to prevent and lower the severity and mortality of COVID-19 disease with novel SARS-CoV-2 variants of concern (VOC).

## Introduction

Novel coronavirus disease 2019 (nCOVID-19) is a severe acute respiratory syndrome coronavirus 2 (SARS-CoV-2) announced as a global pandemic that affected more than 60 million population with more than 6 million deaths to date^1^. At present, the number of Covid-19 cases is still alarming around the world with frequent mutations in the structure of the SARS-COV-2 Virus; hence there is an increasing global concern about this outbreak. Clinical characteristics of COVID-19 manifest as asymptomatic or mild infections in children and young adults, while in older adults, it manifests as severe to critical presentations with acute respiratory distress syndrome (ARDS) and even death^2^. Ppatients with COVID-19 manifest several clinical symptoms as mild, moderate, severe, and critical illness. Initially, COVID-19 present with flu-like symptoms such as high-grade fever, dry cough, and fatigue, followed by dyspnoea^2^.

A spate of recent studies has shown that patients who have pre-existing comorbidities are at increased risk of COVID-19 severity, hospitalization, admission to an intensive care unit, intubation, and mortality compared with those without any comorbidity^3^. In addition, few studies have explored the effect of various comorbidities on the severity of COVID-19 disease that concomitant medical comorbidities such as diabetes, hypertension, chronic kidney diseases, obesity, respiratory diseases and malignancy and age > 50 years were associated with an increased risk of COVID-19 severity, morbidity and mortality^3–8^.

However, as the pandemic progressed, more and more clinical data from all around the world became available to synthesize the updated evidence, to better understand insights into evolving COVID-19 disease severity and to develop strategies for better management of SARS-CoV-2 infected patients. Nonetheless, there is a dearth of data on the prevalence of the most common medical comorbidities that aare associated with increased severity and mortality of the disease such as hypertension, diabetes, obesity, asthma and smoking in patients with COVID-19 among different populations of various continents^2^. Therefore, to address these gaps in the research, the present study was conducted to estimate the prevalence rates and geographical distribution of prior mentioned comorbidities, COVID-19 disease severity and mortality and to evaluate the association between age, gender and smoking status characteristics on hospitalization, ICU admission, and mortality by geographic region, study design among SARS-CoV-2 infected patients from the real-world clinical studies data which will helps to effectively allocate healthcare resources, endorse appropriate preventive and containment measures, and guide emerging treatment protocols .

## Methods

The present systematic review and multistage meta-analyses were reported according to the PRISMA (Preferred Reporting Items for the Systematic Review and Meta-analysis) guidelines.

### Search strategy

A literature search of PubMed/MEDLINE, SCOPUS, Google Scholar and EMBASE was performed from January 2020 to October 2022, using the MESH terms and /or keywords “(Corona Virus Disease-2019) OR (COVID-19) OR (Severe acute respiratory syndrome corona virus 2) OR (SARS-Cov-2) AND (Comorbidities)” with filters for the cross-sectional studies, cohort studies, case series studies, and case–control studies on comorbidities reporting among the COVID-19 populations that were published in English were included. In addition, we searched the reference lists of the relevant publications, reviews and meta-analyses to identify additional potentially relevant studies. Studies with similar authors, the study duration, and the location of the study were strictly matched to further identify any duplicated study. All the duplicates were omitted from the analyses. The search was independently screened by two researchers (SC and VS) and discrepancies were resolved by discussion with a third researcher (SP).

### Study selection

The titles and/or abstracts were reviewed qualitatively by two different authors (CS/MR) reviewed separately to identify studies that evaluated the effect of comorbidities on COVID-19 severity and mortality among hospitalized patients, performed duplicate removal, full-text assessment and discrepancies were resolved through discussion with a third researcher (SP).

### Eligibility criteria

Studies were eligible for inclusion in our systematic review metaanalysis and metaregression study if they met the following criteria: (1) originally published in the English language (2) included confirmed diagnosis of COVID-19 through RTPCR laboratory diagnosis test; (3) provided information about comorbidities; (5) contained information on the disease outcomes: severity or mortality within comorbidity; and (6) published as an original investigation. Studies without diagnostic information, studies that included but did not report comorbidities were excluded from the analysis. When studies did not have available data, we emailed the corresponding authors for information. We excluded studies only if data were not provided at the time of meta-analysis.

### Data extraction

Demographic study characteristics which included first author’s last name, publication year, country and continent (North America, Europe, Asia, Africa, South America) where the research was conducted in, study design, study description or name, study period, the average age in years with standard deviation or interquartile range; status of the comorbidities (number of subjects without any comorbidity, number of subjects with one comorbidity), the type of comorbidity that included hypertension, diabetes mellitus, obesity, asthma, and smoking status, sample size with the number of hospitalizations, ICU admissions and mortality within each comorbid condition. The detail information on the inclusion of comorbidities, outcomes including the criteria for COVID-19 severity assessment, and comparing variables is provided in an additional (Annexure 1). Data were extracted by CS and SP and extractions were checked for accuracy by MC.

### Data analysis

The estimates of obesity, diabetes, hypertension, asthma, smoking, hospitalization rate, ICU admission rate, and mortality rates were expressed as proportions (%) with corresponding 95% confidence intervals (CI). The pooled prevalence estimates of outcome variables were calculated using regional population size weights. The magnitude of heterogeneity between the studies was assessed using the I^2^ statistic (% residual variation due to heterogeneity), and Tau^2^ (method of moments estimate of between-study variance) was used for each of the pooled estimates. I^2^ values range between 0 and 100% and are considered low for I^2^ < 25%, modest for 25–50%, and large for > 50%^9^. As differences between the studies were very high (95–99% inconsistency), a random effect DerSimonian-Laird model was used in all analyses^9^. In case of substantial heterogeneity, the source of heterogeneity was investigated using subgroup analyses based on the study-level characteristics, such as geographical region-wise, study design type, mean age, and women-to-men ratio. The association between the subgroups of each factor was assessed using Cochran’s Q test, degree of freedom(df), and p-value resulting from Cochran’s Q test. A *p* value of < 0.10 was considered statistically significant for Cochran’s Q test (Huedo-Medina et al., 2006). Meta-regression analysis was also performed to find out the strength of the association between age as a moderator and different health conditions. The risk of publication bias was inspected by using the symmetry of funnel plots, and Egger’s and Begg’s tests were also used. Statistical analyses were performed using STATA software, version 16 MP (StataCorp, College Station, TX).

## Results

### Characteristics of all included studies

**Figure 1:**
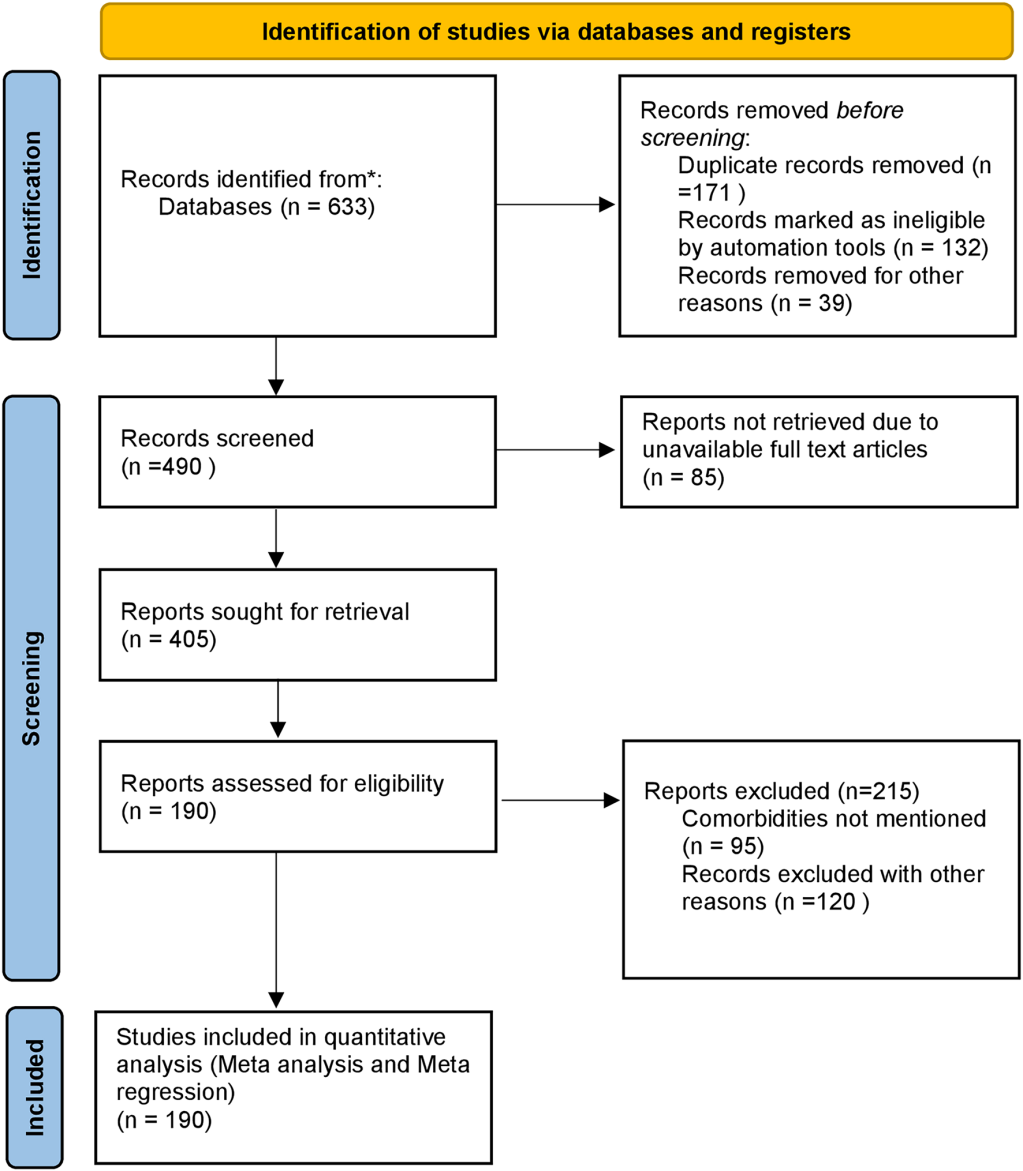
The PRISMA flow diagram for study selection.

All the studies included in the present study were published between January 2020 and 30th October 2022 2021. A total of 647 references were initially identified through electronic databases. After removing duplicates, a total of 490 titles and abstracts were screened to determine if they met the inclusion criteria, as described in the methodology section. Full-text assessment of 405 potentially relevant articles resulted in 190 eligible studies as shown in Fig. 1. Sample size varied on a regional basis from 22 to 55, 86,521, making a total of 1, 05,98, 010 patients. All the studies included both women and men. However, one hundred and thirty-four studies included more men than women. The average age of the study population ranged between 17 and 81 years. Among the included studies, seven were case–control designs and one hundred and eighty-three studies were cohort studies. The majority of the studies were conducted in North America, Seventy-seven in Europe, twenty-three in Asia, six in South America, and one in Africa^10–191^. The characteristics of the included studies are summarized in Annexure 1.

### Prevalence of obesity

**Figure 2:**
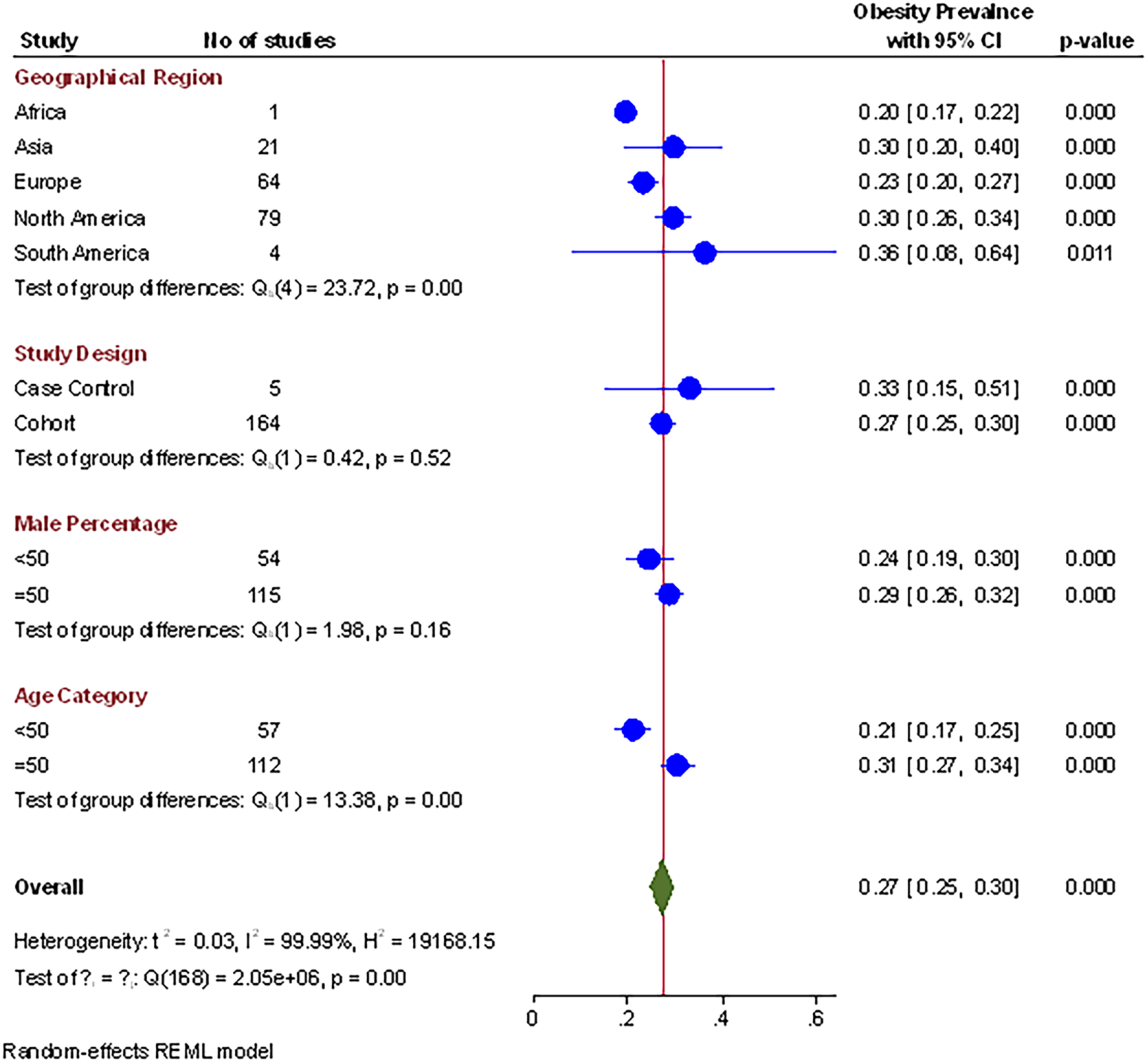
Prevalence of obesity in patients with Covid-19 across various potential variables.

Out of 190 publications, sixty-nine studies, comprising 99, 57,215 participants, reported a prevalence of obesity among covid-19 patients. The pooled prevalence of obesity, after weighing the geographical population size, was 27% (n = 19, 70,472, 95% CI 0.25–0.30, I^2^ = 99.9%, *p* < 0.01, τ^2^ = 0.03), which indicated substantial heterogeneity, as shown in Table 1. Comparison of obesity proportions across the globe showed significant differences (Q = 12.8, *df* = 4; *p* < 0.01). South America, North America, and Asia demonstrated a relatively higher pooled prevalence of 36% (95% CI 8–64, *p* < 0.01), 30%( 95% CI 26–34, *p* < 0.01), and 29% (95% CI 19–39, *p* < 0.01) respectively, while Europe had a lower pooled prevalence of 23% (95% CI 20–26, *p* < 0.01). The variations in the pooled prevalence of obesity are further illustrated in the forest plot in Fig. 2.

**Table 1:** Stratified meta-analysis of the prevalence of different conditions of COVID-19 patients.

Subgroups	No of studies	Prevalence (95% CI)	Test for heterogeneity			Between subgroup differences		
			Tau^2^	I^2^ (%)	*P* value	Q	*df*	*P* value
*Obesity*								
Overall	169	0.27 (0.25 to 0.30)	0.03	99.9	< 0.01			
Geographical Region								
Africa	1	0.20 (0.14 to 0.25	0.001	–	–	12.8	4	0.01
Asia	21	0.29 (0.19 to 0.39)	0.05	95.8	< 0.01			
Europe	64	0.23 (0.20 to 0.26)	0.02	99.7	< 0.01			
North America	79	0.30 (0.26 to 0.34)	0.03	99.9	< 0.01			
South America	4	0.36 (0.08 to 0.64)	0.08	99.2	< 0.01			
Study design								
Case–Control	5	0.31 (0.16 to 0.46)	0.03	99.3	< 0.01	0.3	1	0.57
Cohort	164	0.27 (0.24 to 0.29)	0.03	99.9	< 0.01			
Age (years)								
< 50	57	0.21 (0.17 to 0.24)	0.01	99.9	< 0.01	14.7	1	0.00
≥ 50	112	0.30 (0.27 to 0.33)	0.03	99.7	< 0.01			
Male (%)								
< 50	54	0.24 (0.19 to 0.29)	0.03	99.9	< 0.01	1.8	1	0.18
≥ 50	115	0.28 (0.25 to 0.31)	0.02	99.7	< 0.01			
*Diabetes*								
Overall	175	0.27 (0.25 to 0.30)	0.03	99.9	< 0.01			
Geographical Region								
Africa	1	0.20 (0.17 to 0.23)	0.00	–	–	18.23	4	0.00
Asia	21	0.29 (0.19 to 0.39)	0.01	80.85	< 0.01			
Europe	68	0.20 (0.17 to 0.23)	0.01	99.58	< 0.01			
North America	80	0.27 (0.24 to 0.30)	0.02	99.90	< 0.01			
South America	5	0.29 (0.16 to 0.41)	0.02	96.53	< 0.01			
Study design								
Case–control	7	0.22 (0.16 to 0.46)	0.01	96.10	< 0.01	0.11	1	0.74
Cohort	175	0.23 (0.21 to 0.25)	0.02	99.84	< 0.01			
Age (years)								
< 50	50	0.16 (0.13 to 0.20)	0.02	99.92	< 0.01	23.61	1	0.00
≥ 50	123	0.26(0.24 to 0.29)	0.01	99.45	< 0.01			
Male (%)								
< 50	49	0.17 (0.13 to 0.21)	0.02	99.92	< 0.01	13.12	1	0.00
≥ 50	124	0.26(0.24 to 0.28)	0.01	99.51	< 0.01			
*Hypertension*								
Overall	170	0.39 (0.36 to 0.42)	0.04	99.92	< 0.01			
Geographical Region								
Africa	1	0.41 (0.36 to 0.46)	0.00	–	–	39.88	4	0.00
Asia	20	0.21 (0.15 to 0.27)	0.01	87.34	< 0.01			
Europe	68	0.43 (0.39 to0.47)	0.03	99.80	< 0.01			
North America	74	0.40 (0.35 to 0.45)	0.04	99.96	< 0.01			
South America	6	0.44 (0.24 to 0.63)	0.05	98.77	< 0.01			
Study design								
Case control	7	0.34 (0.22 to 0.45)	0.02	98.94	< 0.01	0.90	1	0.34
Observational	162	0.40 (0.36 to 0.43)	0.04	99.93	< 0.01			
Age (years)								
< 50	49	0.23 (0.18 to 0.27)	0.02	99.94	< 0.01	75.96	1	0.00
≥ 50	118	0.47 (0.43 to 0.50)	0.03	99.69	< 0.01			
Male (%)								
< 50	50	0.32 (0.26 to 0.38)	0.05	99.96	< 0.01	8.31	1	0.00
≥ 50	119	0.42 (0.39 to 0.46)	0.03	99.79	< 0.01			
*Asthma*								
Overall	112	0.08 (0.07 to 0.09)	0.00	98.33	< 0.01			
Geographical Region								
Africa	1	0.05 (− 0.00 to 0.10)	0.00	–	–	58.76	4	0.00
Asia	11	0.07 (0.03 to 0.11)	0.00	57.41	0.01			
Europe	41	0.09 (0.08 to 0.11)	0.00	97.47	< 0.01			
North America	55	0.08 (0.06 to 0.10)	0.00	98.78	< 0.01			
South America	4	0.02 (0.00 to 0.03)	0.00	0.31	0.50			
Study design								
Case control	4	0.05 (0.04 to 0.06)	0.00	0.04	0.82	24.86	1	0.00
Observational	108	0.08 (0.07 to 0.10)	0.00	98.39	< 0.01			
Age (years)								
< 50	43	0.07 (0.05 to 0.09)	0.00	99.21	< 0.01	4.46	1	0.03
≥ 50	69	0.09 (0.08 to 0.11)	0.00	93.89	< 0.01			
Male (%)								
< 50	35	0.09 (0.06 to 0.11)	0.00	97.92	< 0.01	0.11	1	0.74
≥ 50	77	0.08 (0.07 to 0.09)	0.00	98.23	< 0.01			
*Smoking*								
Overall	99	0.15 (0.12 to 0.18)	0.02	99.83	< 0.01			
Geographical Region								
Africa	1	0.07 (0.02 to 0.12)	0.00	–	–	7.27	3	0.06
Asia	12	0.16 (0.07 to 0.24)	0.02	83.00	< 0.01			
Europe	38	0.16 (0.11 to 0.22)	0.03	99.88	< 0.01			
North America	48	0.14 (0.10 to 0.18)	0.01	99.80	< 0.01			
Study design								
Case control	6	0.13 (0.08 to 0.18)	0.00	92.42	< 0.01	0.69	1	0.41
Observational	93	0.15 (0.12 to 0.18)	0.02	99.85	< 0.01			
Age (years)								
< 50	35	0.11 (0.07 to 0.16)	0.02	99.87	< 0.01	4.25	1	0.04
≥ 50	64	0.17 (0.14 to 0.21)	0.02	99.70	< 0.01			
Male (%)								
< 50	29	0.12 (0.08 to 0.16)	0.01	99.73	< 0.01	2.38	1	0.12
≥ 50	70	0.16 (0.12 to 0.20)	0.02	99.82	< 0.01			
*Hospitalized*								
Overall	61	0.35 (0.29 to 0.41)	0.00	99.96	< 0.01			
Geographical Region								
Africa	1	0.47 (0.42 to 0.53)	0.00	–	–	69.94	4	0.00
Asia	2	0.31 (0.13 to 0.49)	0.02	90.58	< 0.01			
Europe	17	0.35 (0.18 to 0.510	0.12	99.96	< 0.01			
North America	40	0.35 (0.29 to 0.41)	0.04	99.97	< 0.01			
South America	1	0.57(0.55 to 0.58)	0.00	–	–			
Study design								
Case control	3	0.42 (0.19 to 0.65)	0.04	99.76	< 0.01	0.36	1	0.00
Observational	58	0.35 (0.29 to 0.41)	0.06	99.98	< 0.01			
Age (years)								
< 50	36	0.31 (0.25 to 0.37)	0.03	99.97	< 0.01	2.53	1	0.11
≥ 50	25	0.41 (0.30 to 0.53)	0.08	99.97	< 0.01			
Male (%)								
< 50	32	0.31 (0.23 to 0.39)	0.06	99.98	< 0.01	2.22	1	0.00
≥ 50	29	0.40 (0.32 to 0.48)	0.05	99.96	< 0.01			
*ICU Admission*								
Overall	106	0.17 (0.14 to 0.21)	0.03	99.85	< 0.01			
Geographical Region								
Africa	1	0.06 (0.01 to 0.11)	0.00	–	–	27.57	4	0.00
Asia	15	0.18 (0.08 to 0.28)	0.03	95.01	< 0.01			
Europe	37	0.18 (0.12 to 0.25)	0.04	99.78	< 0.01			
North America	52	0.16 (0.12 to 0.20)	0.02	99.85	< 0.01			
South America	1	0.20 (0.19 to 0.22)	0.00	–	–			
Study design								
Case control	5	0.13 (0.04 to 0.22)	0.01	98.35	< 0.01	0.95	1	0.33
Observational	101	0.18 (0.14 to 0.21)	0.03	99.86	< 0.01			
Age (years)								
< 50	45	0.13 (0.08)	0.02	99.90	< 0.01	5.12	1	0.02
≥ 50	61	0.20 (0.16)	0.03	99.53	< 0.01			
Male (%)								
< 50	37	0.14 (0.09 to 0.19)	0.02	99.60	< 0.01	2.16	1	0.14
≥ 50	69	0.19 (0.15 to 0.23)	0.03	99.88	< 0.01			
*Mortality*								
Overall	145	0.18 (0.16 to 0.21)	0.04	98.65	< 0.01			
Geographical Region								
Africa	1	0.11 (0.06 to 0.16)	0.00	–	–	19.04	4	0.00
Asia	9	0.08 (0.01 to 0.14)	0.01	84.63	< 0.01			
Europe	61	0.20 (0.16 to 0.23)	0.02	99.58	< 0.01			
North America	72	0.18 (0.14 to 0.21)	0.02	99.93	< 0.01			
South America	4	0.37 (0.17 to 0.56)	0.04	98.65	< 0.01			
Study design								
Case control	6	0.14 (0.07 to 0.21)	0.01	96.80	< 0.01	1.40	1	0.00
Observational	141	0.19 (0.16 to 0.21)	0.02	99.89	< 0.01			
Age (years)								
< 50	36	0.09 (0.06 to 0.13)	0.01	99.91	< 0.01	28.84	1	0.00
≥ 50	111	0.22 (0.19 to 0.24)	0.02	99.58	< 0.01			
Male (%)								
< 50	44	0.12 (0.08 to 0.16)	0.02	99.87	< 0.01	15.12	1	0.00
≥ 50	101	0.21 (0.18 to 0.24)	0.02	99.77	< 0.01

### Prevalence of diabetes

One hundred and seventy-five studies investigated the prevalence of diabetes among Covid-19 patients. The pooled estimate of diabetes was 27% (n = 9, 56,475, 95% CI 0.25–0.30, I^2^ = 99.9%, *p* < 0.01, τ^2^ = 0.03), which indicated substantial heterogeneity, as shown in Table 1. Comparison of diabetes prevalence across the world showed significant differences (Q = 18.2, *df* = 4; *p* < 0.01). South America and Asia have shown a higher pooled prevalence of 29% each, followed by North America with 27% (95% CI 0.24–0.30, I^2^ = 99.9%). Whereas Europe has demonstrated a relatively lower pooled prevalence of 20% (95% CI 17–23, *p* < 0.01).

### Prevalence of hypertension

Among included studies, one hundred and seventy studies provided hypertension estimates among Covid-19 patients. The pooled prevalence of hypertension was found to be 39% by using the random effects model (n = 17, 68,567, 95% CI 0.36–0.42, I^2^ = 99.9%, *p* < 0.01, τ2 = 0.04), which indicates considerable heterogeneity as shown in Table 1. Comparison of hypertension proportions across the globe showed significant differences (Q = 39.88, *df* = 4; *p* < 0.01). South America, Europe and North America demonstrated a relatively higher pooled prevalence of 44% (95% CI 24–63, *p* < 0.01),43% (95% CI 39–47, *p* < 0.01), and 40% (95% CI 35–45, *p* < 0.01)respectively, while the Asiatic region had a lower pooled prevalence of 21%( 95% CI 15–27, *p* < 0.01).

### Prevalence of asthma

Out of 190 publications, a total of 112 studies reported the prevalence of asthma in patients with Covid-19. The pooled prevalence of asthma, after weighing the geographical population size, was 8% (n = 1, 75,177, 95% CI 0.7–0.9, I^2^ = 98.3%, *p* < 0.01, τ^2^ = 0.00), which indicated substantial heterogeneity, as shown in Table 1. Comparison of asthma proportions across the globe showed significant differences (Q = 58.7, *df* = 4; *p* < 0.01). Europe, North America, and Asia demonstrated a relatively higher pooled prevalence of 9% (95% CI 8–11, *p* < 0.01), 8% (95% CI 6–10, *p* < 0.01), and 7% (95% CI 3–11, *p* < 0.01)respectively, while South America had a lower pooled prevalence of 2% (95% CI 0.0–03, *p* = 0.05).

### Prevalence of smoking

Ninety-nine studies investigated the prevalence of smoking in patients with Covid-19. The pooled estimate of smoking was 15% (n = 4, 41, 809, 95% CI 0.12–0.18, I^2^ = 99.9%, *p* < 0.01, τ^2^ = 0.03), which indicated substantial heterogeneity, as shown in Table 1. Comparison of smoking prevalence across the world showed non-significant differences (Q = 7.2, *df* = 3; *p* = 0.06). Europe and Asia have shown a higher pooled prevalence of 16% (95% CI 11–22, *p* < 0.01) and 16% (95% CI 07–24, *p* < 0.01). While North America with 14% (95% CI 10–18, *p* < 0.01).

### Prevalence of hospitalization

Out of 195 publications, a total of 61 studies reported the prevalence of hospitalization in patients with Covid-19. The pooled prevalence of hospitalization, after weighing the geographical population size, was 35% (n = 7, 48,526, 95% CI 0.29–0.41, I^2^ = 99.9%, *p* < 0.01, τ^2^ = 0.00), which indicated substantially heterogeneity, as shown in Table 1. Comparison of hospitalization proportions across the globe showed significant differences (Q = 69.9, *df* = 4; *p* < 0.01). South America demonstrated a relatively higher pooled prevalence of 57% (95% CI 0.55–0.58, *p* < 0.01), while Asia had pooled prevalence of hospitalization of 31% (95% CI 13–49, *p* < 0.01).

### Prevalence of ICU admissions

One hundred and six studies investigated the prevalence of ICU admissions among Covid-19 patients. The pooled estimate of ICU admissions was 17% (n = 1, 93,980, 95% CI 0.14–021, I^2^ = 99.8%, *p* < 0.01, τ^2^ = 0.03), which indicated substantial heterogeneity, as shown in Table 1. Comparison of ICU admission prevalence across the world showed significant differences (Q = 27.5, *df* = 4; *p* < 0.01). South America has showed a higher pooled prevalence of 20% (95% CI 0.19–0.22), followed by Asia and Europe with 18% (95% CI 0.08—0.28), 18% (95% CI (0.12–0.25) of each. Whereas North America has shown a lower pooled prevalence of 16% (95% CI 0.12–0.20).

### Prevalence of mortality

Out of 195 publications, a total of 145 studies reported the prevalence of mortality rate in patients with Covid-19. The pooled prevalence of mortality, after weighing the geographical population size, was 18% (n = 4, 45,854, 95% CI 0.16–0.21, I^2^ = 98.3%, *p* < 0.01, τ^2^ = 0.00), which indicated substantially heterogeneity, as shown in Table 1. Comparison of mortality proportions across the globe showed significant differences (Q = 19.04, *df* = 4; *p* < 0.01). South America, Europe, and North America demonstrated a relatively higher pooled prevalence of 37% (95% CI 0.17–56, *p* < 0.01), 20% (95% CI 0.16–0.23, *p* < 0.01), and 18% (95% CI 0.14–0.21, *p* < 0.01)respectively, while Asia had a lower pooled prevalence of 8% (95% CI 0.01- 0.14).

### Subgroup analysis

Subgroup analysis by geographic region, study design, age category, and male percentage did not influence the prevalence estimates of obesity, diabetes, hypertension, asthma, smoking, hospitalization, ICU admissions, and mortality rate as shown in Table 1. However, the prevalence of mortality (12%), ICU admission rate (14%), and hospitalization rate (31%) was low among patients with ages < 50 years. The prevalence of each condition was high in the > 50 years age male population as compared to the < 50 years age male population, except in Asthma.

### Meta-regression analysis

**Figure 3:**
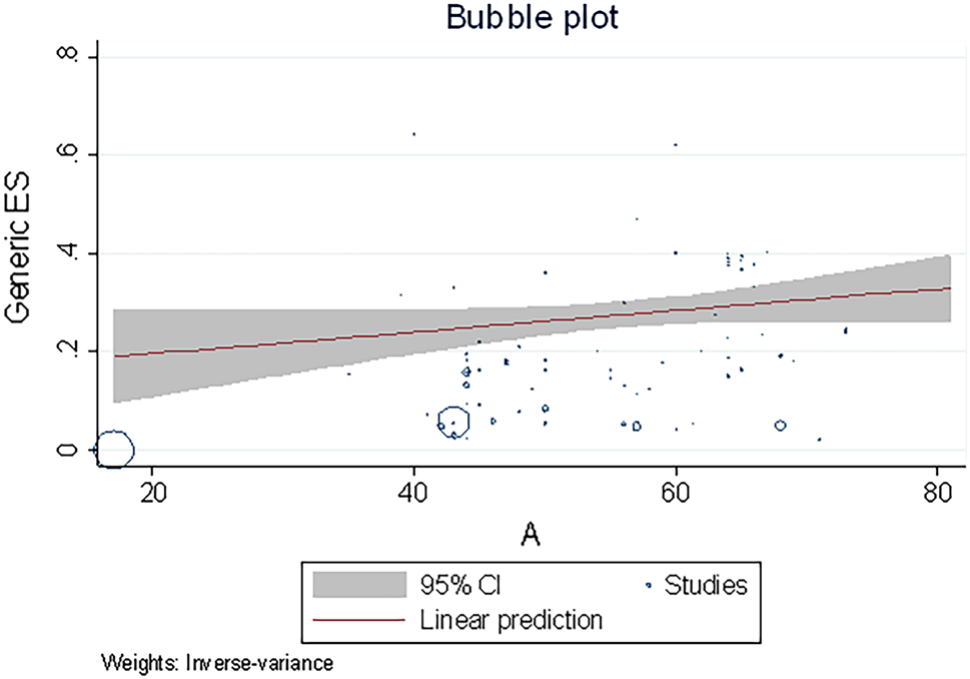
Regression for the prevalence of diabetes according to the age in patients with Covid-19.

Meta-regression suggested a statistically significant relationship between the prevalence of hospitalization and smoking (*p* = 0.04, asthma (*p* = 0.03), obesity (*p* = 0.03), and hypertension (*p* = 0.02) (Table 2). However, the prevalence of mortality showed no relationship between all the comorbidity conditions, smoking and hospitalization (Table 3). Meta-regression suggested a statistically significant relationship between age and diabetes (*p* < 0.001, Fig. 3), hypertension (*p* < 0.001), asthma (*p* < 0.05), ICU admission rate (*p* < 0.05), and mortality (*p* < 0.001). However, there was no evidence of a relationship between age and obesity (*p* = 0.07), smoking (*p* = 0.11), and hospitalization (*p* = 0.057) as shown in Table 4.

**Table 2:** Meta-regression between prevalence of hospitalization and different conditions as moderator.

Condition	Coefficient	SE	Z	95% CI	*P* value
Obesity	− 4.94	2.29	− 2.16	− 9.42 to − 4.53	0.03
Diabetes	− 1.25	6.92	− 1.81	− 2.61 to 1.03	0.07
Hypertension	− 7.29	3.23	− 2.26	− 1.36 to − 9.60	0.02
Asthma	− 8.41	3.92	− 2.07	− 0.0001 to − 4.72	0.03
Smoking	− 2.67	1.34	− 2.00	− 5.29 to − 4.72	0.04
ICU Admission	− 4.59	6.07	− 0.08	− 0.0001 to 0.0001	0.94
Mortality	− 3.27	2.45	− 1.33	− 8.07 to 1.54	0.18

*SE* standard error, *CI* confidence interval.

**Table 3:** Meta-regression between prevalence of mortality and different conditions as moderator.

Condition	Coefficient	SE	Z	95% CI	*P* value
Obesity	− 6.45	6.91	− 0.93	− 2.00 to 7.10	0.35
Diabetes	− 5.80	1.95	− 0.30	− 4.41 to 3.25	0.76
Hypertension	− 7.34	9.23	− 0.80	− 2.54 to 1.07	0.42
Asthma	1.48	1.63	0.91	− 1.71 to 4.68	0.36
Smoking	7.66	8.07	0.95	− 8.15 to 2.35	0.34
ICU Admission	− 9.17	1.27	0.72	− 1.57 to 3.40	0.46
Hospitalization	2.92	3.46	5.69	− 3.86 to 9.71	0.39

*SE* standard error, *CI* confidence intervel.

**Table 4:** Meta-regression between different conditions and age as moderator.

Condition	Coefficient	SE	Z	95% CI	*P* value
Obesity	0.002	0.001	1.79	− 0.0002 to 0.004	0.07
Diabetes	0.005	0.0009	5.77	0.003 to 0.007	0.000
Hypertension	0.012	0.001	11.04	0.10 to 0.146	0.000
Asthma	0.001	0.0004	2.40	0.0002 to 0.002	0.016
Smoking	0.002	0.001	1.58	− 0.005 to 0.004	0.113
Hospitalised	0.006	0.003	1.90	− 0.0001 to 0.012	0.057
ICU Admission	0.003	0.001	2.08	0.0001 to 0.006	0.037
Mortality	0.006	0.001	5.22	0.0039 to 0.008	0.000

*SE* standard error; *CI* confidence.

### Publication bias assessment

**Figure 4:**
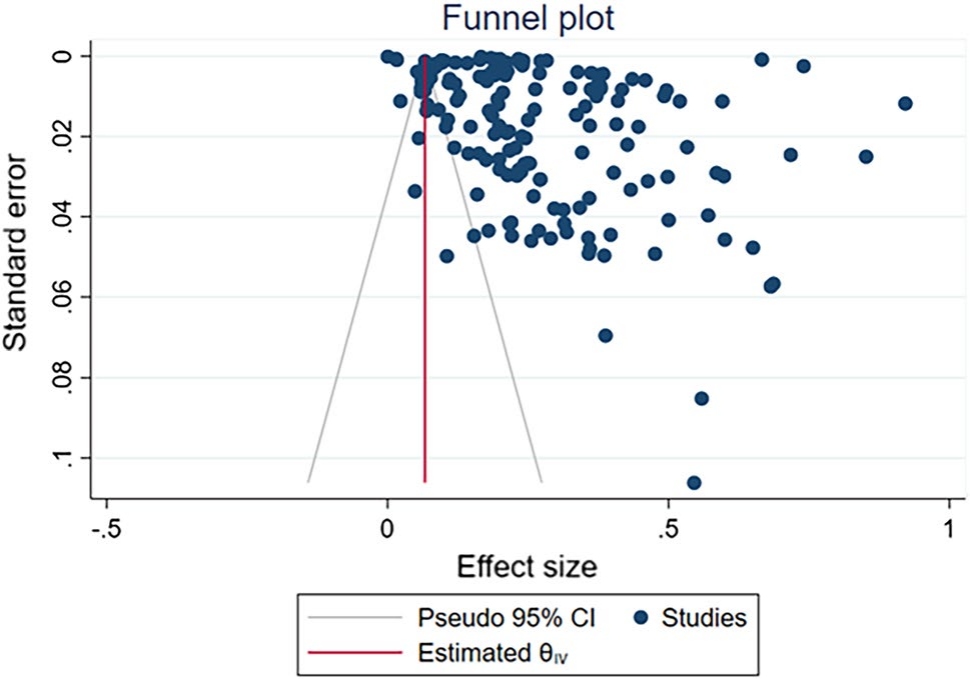
Funnel plot of the effect size on prevalence of obesity in patients with Covid-19.

The Egger’s and Begg’s tests indicated statistically significant publication bias for the estimates of obesity(Egger test: *p* < 0.0001 & Begg’s test:* p* < 0.0001, Fig. 4), diabetes (Egger test:* p* < 0.0001&Begg’s test:* p* < 0.0001), hypertension (Egger test: *p* < 0.0001&Begg’s test:* p* < 0.0001), asthma (Egger test: *p* < 0.005 & Begg’s test: *p* < 0.07), smoking (Egger test: *p* < 0.005 & Begg’s test:* p* < 0.0001), hospitalization (Egger test: *p* = 0.12 & Begg’s test: *p* < 0.0001), ICU admissions (Egger test: *p* < 0.0001 & Begg’s test:* p* < 0.0001), and mortality rate (Egger test:* p* < 0.0001 & Begg’s test:* p* < 0.0001). Visual examination of the funnel plots showed symmetry and suggested no publication bias, as shown in Fig. 4.

## Discussion

To our knowledge, the present study is one of the largest meta-analyses of the global prevalence of the most common comorbidities such as diabetes, hypertension, obesity, asthma, and meta-regression of the association between age, gender, smoking status and hospitalization, ICU admissions, and mortality in patients with COVID-19. In addition to estimating the prevalence of common comorbidities, the present study results also revealed some new insights into novel corona disease 2019 in the current era of the ongoing pandemic. The present study estimated the highest and lowest proportions of the four most common comorbidities in patients with COVID-19 from different geographic regions from real-world studies. The prevalence of obesity was higher in South America, followed by Europe and Asia, Diabetes in South America, North America and Europe, Hypertension in South America, Europe, Africa and North America, Asthma in Europe, North America and Asia, Smoking in Europe, Asia and North America. Although the prevalence of obesity, diabetes, and hypertension was higher in South America, it is not possible to comment on the prevalence of all three comorbidities in South America due to a wide 95% confidence interval (due to a smaller number of studies), making this a wise decision. Overall, the prevalence of various comorbid conditions in patients with COVID-19 was highest in North America, Europe, and Asia, while both South America and Africa had a lower prevalence of all four major comorbidities. The most common reason for an increased prevalence of these comorbidities in North America, Europe and Africa might be due to the large number of studies published in South America and Africa. Findings of the prevalence rate of hospitalization among patients with COVID-19 had shown a significant difference across the globe (*p* < 0.01). Both North America and Europe have demonstrated a relatively higher prevalence of hospitalization, followed by Asia, South America and Africa. The prevalence rate of ICU admission was higher with severe COVID-19, was higher in Europe, North America and Asia. The prevalence of mortality among COVID-19 patients was higher in Europe, North America and Asia. Overall Prevalence of each comorbidity was more among the > 50 years age group population than < 50 years and in males, as compared to the < 50 years age group and female population, except for Asthma. In our present study, the prevalence of hospitalization, ICU admission and mortality rate were lower in patients < 50 years of age, than in patients > 50 years of age group, with a higher prevalence of concomitant comorbidities. The prevalence of comorbidities might be the cause of increased mortality among patients in the > 50 years of age group than the < 50 years of age group. Globally, the relationship between age and comorbidities diabetes, hypertension, asthma, ICU admission rate, and mortality has been shown as significant relation.

A spate of previous meta-analysis studies has shown that pre-existing diabetes, hypertension, obesity and smoking were associated with higher mortality associated with COVID-19 a total of nearly 30%^192–196^. In patients with diabetes mellitus, hyperglycaemia-associated causes modify immunological and inflammatory processes, predisposing individuals to severe, potentially fatal COVID-19^196^. Obesity is associated with significant changes in the distribution and number of immune cells in the adipose tissues, with fewer Treg cells, Th2 cells, and M2 macrophages, which will cause cells to decrease in quantity, especially M1 macrophages and CD8 + T cells increases, in similar with autoimmune diseases^196,197^. Therefore, obesity affects the immune defence and T cell activity^196,197^. Overall, comorbidities such as Hypertension, diabetes mellitus, obesity and smoking are significantly associated with vascular endothelial injury, dysfunctional haemostatic system, and pro-inflammatory or chronic inflammation state, leading to cytokine storm, multi-organ failure (MOF) and acute respiratory distress syndrome (ARDS) ^197–199^. This relationship was further supported in a recent study, which showed that the male gender and elderly ages were associated with higher morbidity or mortality due to COVID-19^200^. While former smokers appear to be at increased risk of hospitalization, increased disease severity and mortality from COVID-19 than never smokers and current smokers^201^. However, this relationship was further supported in a recent study, which showed that asthma as co-morbidity doesn’t have a significant risk of SARS-CoV-2 infection, severity and mortality with COVID-19^201–204^.

There are several limitations in the present systematic review and meta-analysis. First, most of the included studies had observational (prospective and retrospective) study design heterogeneity of studies was observed in the analyses of continuous variables. In addition, only studies in the English language were included in the present study. Moreover, there were a smaller number of studies found in the geographic regions of Africa and South America, whereas the majority of studies were from North America, Europe, and Asia which further increases the possibility of publication bias.

## Conclusion

In this systematic review, metaanalysis and metaregression study, an overall higher prevalence of hypertension (39%), diabetes(27%), obesity (27%), and 18% of mortality  among hospitalized patients with COVID-19 across the world. Geographic regions with a higher pooled prevalence of comorbidities, specifically, North America, and Europe, had shown a high prevalence estimates of all the major comorbid conditions and mortality followed by South America, Asia and Africa. The present meta-analysis and meta-regression will help to make an appropriate decisions by administrators, stakeholders and health care providers to take a clinical decision among patients with comorbidities and to be vigilant over disease severity and mortality in relation to smoking status, age and gender wise. We suggest for regular booster dose vaccination preferably for those patients with chronic comorbidities and to follow regular preventive measures to contain the spread of highly infectious novel  variants of SARS-CoV-2  omicron variants and to prevent the severety, mortality of COVID-19 disease.

## Supplementary Information

Supplementary Information.

## Data Availability

The datasets used and/or analysed during the current study available from the corresponding author on reasonable request.
